# The *C. elegans* WASH complex supports epithelial polarity by promoting endosomal sorting of E-Cadherin

**DOI:** 10.1091/mbc.E25-12-0612

**Published:** 2026-06-11

**Authors:** Patricia Irizarry-Barreto, Jennifer Smolyn, Victoria Brown, Bhagavathi Ramamurthy, Martha C. Soto

**Affiliations:** ^a^Department of Pathology and Laboratory Medicine, RWJMS, Rutgers University, Piscataway, NJ 08854; ^b^Department of Physician Assistant Studies and Practice, SHP, Rutgers University, Piscataway, NJ 08854; Molecular Biology of the Cell

## Abstract

Epithelial polarity requires polarized distribution of the apical adhesion complex that connects cells through the E-Cadherin transmembrane protein. E-Cadherin is intimately linked to the actin cytoskeleton through alpha catenin, which directly binds F-actin to set up the apical actin belt. Branched actin is formed when the Arp2/3 complex is activated by nucleation-promoting factors (NPFs). *C. elegans* has three NPFs, WASP, WAVE, and WASH. Our studies showed that WAVE-dependent branched actin promotes apical transport of E-Cadherin, including apically-directed transport of RAB-11-enriched endosomes. However, the contribution of other NPFs to E-Cadherin polarity has not been examined. The *C. elegans* WASH complex is not well described. Here we characterize components of the WASH complex, and provide evidence that CO5G5.2, despite being highly divergent, is the functional WSHC-2/FAM21 component in *C. elegans*. We show that the WASH complex is enriched at early and recycling endosomes in the adult intestine, where it supports retrograde E-Cadherin transport. Our findings demonstrate that individual branched actin regulators promote specific transport steps and identify WASH function at RME-1/EHD-enriched endosomes as an important contributor to E-Cadherin polarity and cargo sorting in a mature epithelium.

## Abstract (executive summary)

- *C. elegans* has three known NPF complexes to activate Arp2/3: WAVE, WASP, and WASH. However, the role of *C. elegans* WASH complex in intracellular transport was unreported, and the essential component, WSHC-2/FAM21, was not identified.
- We show that *C. elegans* C05G5.2 is a likely WSHC-2/ FAM21 ortholog, since it shares structural features with WSHC-2 orthologs. Loss of WASH components, including the WSHC-2 ortholog, altered retrograde transport and reduced epithelial polarity, while the cargo -E-Cadherin was mis-sorted from early endosomes.
- WASH-branched actin polarizes epithelia to promote sorting of retrograde cargo, including E-Cadherin.

## INTRODUCTION

**FIGURE 1:**
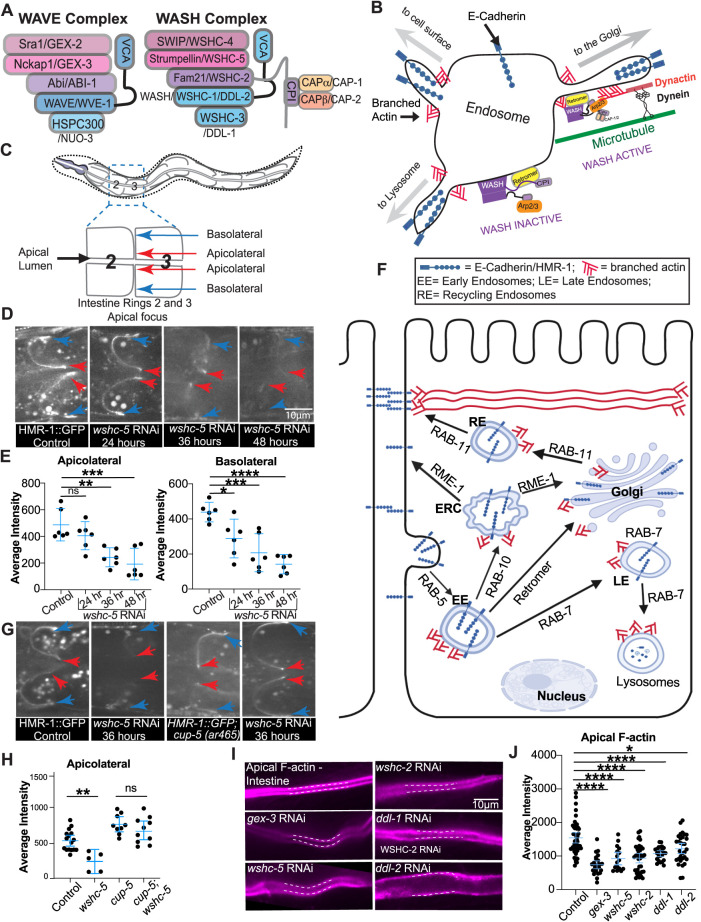
*C. elegans* WASH complex supports E-Cadherin enrichment and apical polarity. (A) The WAVE and WASH complexes, which promote branched actin as nucleation-promoting factors (NPFs) for Arp2/3, have a similar structure. WAVE and WASH are composed of five proteins, including the paralogs WAVE/WVE-1 and WASH/WSHC-1/DDL-2, which activate Arp2/3 with their VCA (verprolin central acidic) domains. (B) Model of WASH complex (purple) on an endosome, highlighting its interactions with Retromer (yellow) at the endosomal membranes and with the Dynactin/Dynein complex, on microtubules. The interaction with Dynactin activates WASH-dependent branched actin that is proposed to support vesicle tubulation and scission (see also 1F). (C) Cartoon of *C. elegans* intestine with a focus on Rings 2 and 3, composed of two cells each, where all measurements were done. Rings 2/3 are behind the pharynx and before the germline, making live imaging simple. Images in most figures are shown from an apical focus, unless otherwise stated, which allows comparison of protein enrichment at apical lumen, apicolateral (red arrows), and basolateral (blue arrows) membrane regions. (D and E) RNAi depletion of *wshc-5* was monitored for effects on E-Cadherin/HMR-1::GFP. Each dot represents one cell–cell junction measurement. *N* = 6 cells. (F) Model for how WASH-dependent branched actin may support HMR-1/E-Cadherin (blue) transport in epithelia. WASH is predicted to support retromer transport, from early endosomes (EE) to the Golgi. Figure 1F, except for some labeling, was created in BioRender. Irizarry-Barreto, P. (2026) https://BioRender.com/wbqcrgs (G and H) HMR-1::GFP strain from D in controls and animals with reduced *wshc-5, cup-5*, and the *wshc-5(RNAi) cup-5(ar465)* double, measured as in D and E. Each dot represents one cell-cell junction. *N* = at least 5 cells. (I and J) Intestinal apical F-actin was measured using strain OX966 *Pglo-1-::Lifeact-Tag-RFP*, in controls and animals depleted of *gex-3*, *wshc-5, wshc-2, ddl-1,* and *ddl-2* using RNAi for 48 h. Each dot represents one line measurement of 10 um along the ring 2 and 3 lumen, four lines per worm, *N* = 5 worms. Statistical analyses here and elsewhere used one-way ANOVA, unless stated otherwise. Asterisks here and in all figures mark statistical significance: *p < 0.05, **p < 0.01, ***p < 0.001, ****p < 0.0001. Here and in all figures, the central lateral blue bar shows the Mean, while vertical blue bars show 95% confidence intervals.

*C. elegans* has three nucleation-promoting factors (NPFs) WASP, WAVE, and WASH, that can activate the Arp2/3 complex, promoting branched actin formation. Branched actin promotes movements and dynamics of cellular events, it plays a large role in protein transport. The NPF WASP supports endocytosis in yeast, mammals, and *C. elegans* (Engqvist-Goldstein and Drubin, 2003; Benesch *et al.*, 2005; Giuliani *et al.*, 2009). Our work showed a role for WAVE in transport (Giuliani *et al.*, 2009; Patel *et al.*, 2013), including apically directed RAB-11 transport, and WAVE accumulation at the Golgi (Cordova-Burgos *et al.*, 2023). The discovery of WASH was accompanied by findings that it has an important role at endosomes (Derivery *et al.*, 2009; Gomez and Billadeau, 2009; Romano-Moreno *et al.*, 2024). WASH is unique among the highly conserved Arp2/3 NPFs, since it directly links membrane dynamics with actin and microtubule (MT) cytoskeletons (Figure 1, A and B). In *Drosophila*, WASH bundled MTs, but WASP and SCAR/WAVE could not (Liu *et al.*, 2009). Since invertebrates like *C. elegans, Dictyostelium,* and *Drosophila* do not have orthologs of the NPF WHAMM (Campellone *et al.*, 2008), WASH may work in invertebrates to sort proteins at Golgi-ER (Gomez and Billadeau, 2009).

WASH is the least studied of the three NPFs in *C. elegans*. Since the five components of the WASH complex have multiple names across species, we refer to the *C. elegans* WASH complex orthologs by their WSHC1-5 name, and where it differs, also by their *C. elegans* name. Two components of the *C. elegans* WASH complex, WSHC-1/*ddl-2*/WASH and *WSHC-3/ddl-1*/CCDC53, were named “*ddl*” as *daf-16*-dependent longevity genes in a genome-wide RNAi screen (Hansen *et al.*, 2005). WSHC-1/DDL-2 and WSHC-3/DDL-1 were shown to regulate heat-shock transcription factor HSF-1 to modulate longevity and thermotolerance (Chiang *et al*., 2012). A third WASH component, WSHC-5/Strumpellin, was CRISPR tagged at its endogenous locus and shown to localize in puncta in embryos (Zhang *et al.*, 2025). A Master's Thesis from our lab reported the first characterization of WASH in *C. elegans* transport (Smolyn, 2020).

WSHC-1/DDL-2 was identified by proximity labeling and genetic experiments as a likely target of the NEKL-MLT kinase complexes that are involved in membrane trafficking and actin regulation (Fay *et al.*, 2026).

Protein transport of adhesion molecules is essential for the formation and maintenance of healthy junctions (Brüser and Bogdan, 2017). E-Cadherin transport was proposed to require actin (Woichansky *et al.*, 2016), but the actin regulators have only recently been identified, including WAVE, which supports apically directed transport on RAB-11 endosomes (Cordova-Burgos *et al.*, 2023). WAVE also supports endosomal transport of yolk proteins (Giuliani *et al.*, 2009) and transport of Wls/MIG-14, TGN-38, and E-Cadherin retrograde recycling from recycling endosomes to the Golgi (Bai and Grant, 2015; Cordova-Burgos *et al.*, 2023). The role of WASH in E-Cadherin transport has not been investigated.

## RESULTS AND DISCUSSION

### *C. elegans* WASH complex supports apical enrichment of E-Cadherin

Our previous studies demonstrated that WAVE supported E-Cadherin transport. However, WAVE was not enriched at all endosomal organelles and did not alter E-Cadherin enrichment at all endosomal organelles, suggesting other branched actin regulators may be involved. We previously found that loss of WASP had minor effects on epithelial transport (Patel *et al.*, 2013). However, WASH, a protein complex with striking similarities to the WAVE complex (Figure 1, A and B), had not been examined for effects on E-Cadherin.

We monitored the distribution of E-Cadherin, a transmembrane protein that is transported with the help of WAVE-branched actin, but which has not been examined for transport by WASH.

Endogenously tagged E-Cadherin/HMR-1::GFP (Marston DJ *et al.,* 2016) is normally enriched at the apicolateral regions of epithelia, like adult intestine, with lower enrichment at more basolateral regions (Cordova-Burgos, 2021, 2023; Figure 1C). To test if E-Cadherin enrichment depended on WASH, we depleted the WASH component *wshc-5* using RNAi (as in Cordova-Burgos *et al.*, 2023). Loss of any core WASH component (*wshc-2, wshc-5,* or *wshc-4/SWIP*) is expected to destabilize the WASH complex (Jia *et al.*, 2010). Depleting *wshc-5*/Strumpellin for 24 h showed mild reduction of E-Cadherin/HRMI-1::GFP levels, 36 h depletion showed stronger reduction, while 48 h depletion strongly reduced E-Cadherin/HMR-1::GFP levels, making it difficult to detect (Figure 1, D and E).

To test if the loss of Cadherin is due to Cadherin mis-sorting to the lysosome, which would increase Cadherin degradation, we tested Cadherin levels in animals defective for lysosome function. While loss of *wshc-5* resulted in significantly decreased apicolateral cadherin (Figure 1, D and G), blocking lysosome function with a mutation in *cup-5* (Fares and Greenwald, 2001a, b), the orthologue of mammalian TRPML1, slightly increased apicolateral cadherin (Figure 1, G and H). A strain missing both *cup-5* and *wshc-5* resembled the *cup-5* single mutant, partially rescuing the loss of apicoleteral E-cadherin (Figure 1, G and H). This result suggested WSHC-5 promotes protein transport.

To directly visualize the consequences of E-Cadherin apicolateral loss, we imaged F-actin using Lifeact expressed in the intestine (Cordova-Burgos *et al.,* 2021) and compared controls to animals depleted of *wshc-5* and WAVE component *gex-3* for 36 h. As previously shown, loss of *gex-3* significantly decreased apical F-actin in the adult intestine. Loss of *wshc-5, wshc-2, ddl-1,* or *ddl-2* also decreased apical F-actin (Figure 1, I and J). We previously showed that depletion of E-Cadherin/HMR-1 results in reduced apical F-actin (Bernadskaya et al., 2011, Cordova-Burgos *et al.,* 2021). Thus, the reduction in E-Cadherin caused by depleted WASH complex is likely to contribute to reduced apical F-actin, which suggests reduced epithelial polarity of the adult intestine (Figure 1, G–J).

### WASH supports the cellular organization of endosomal organelles

WASH is expected to be enriched at endosomes (Derivery *et al.*, 2009; Gomez and Billadeau, 2009; Duleh and Welch, 2010; Romano-Moreno *et al.*, 2024; Figure 1F). To understand what occurs at distinct endosomal organelles when WASH components are depleted, we compared control animals with animals depleted of *wshc-5* for the patterns of early endosomes enriched in RFP::RAB-5, late endosomes enriched for RFP::RAB-7, recycling endosomes enriched for RFP::RME-1 or RFP::RAB-10, and the Golgi, where AMAN-2::GFP is enriched (Treusch *et al.*, 2004; Gleason *et al.*, 2016).

**FIGURE 2:**
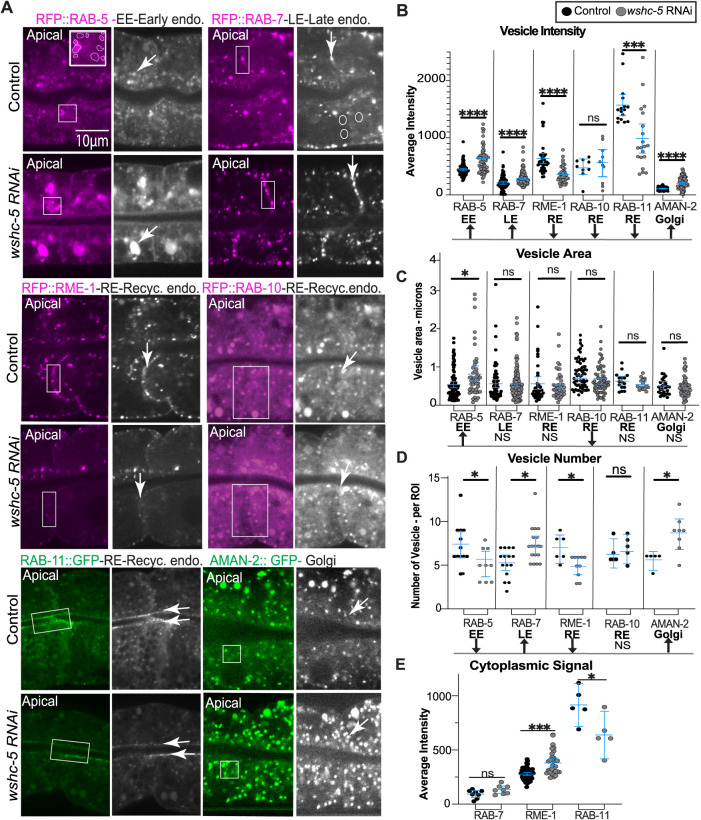
WASH supports cellular organization of endosomal organelles. (A) Apical views of intestinal Rings 2/3 are shown for each endosomal organelle. The top images are controls, and immediately below are animals depleted of *wshc-5* (RNAi, 36 h). The white boxes indicate the region where measurements were made for each endosome. The larger box in the RFP::RAB-5 control illustrates the freehand curves drawn around puncta within the boxes to compare vesicle intensity, vesicle area, and vesicle number, in equally sized regions of individual worms for B, C, and D. For RFP::RAB-10, we averaged the region in the larger box. White arrows indicate regions that were compared. Scale bar = 10 µm. Each dot in B and C = one measured puncta, from at least four animals per genotype. Each dot in D is the number of vesicles in a 50 µm by 50 µm region. For (E), small circles were sampled in cytoplasmic regions, two to three circles per ring, *N* = at least five rings. RAB-5 (*n* = 8); RAB-7 (*n* = 4); RAB-10 (*n* = 5); RME-1 (*n* = 5); AMAN-2 (*n* = 5); RAB-11 (*n* = 5). Statistical analyses in B–E used an unpaired *t* test, with the Welch's correction.

Loss of *wshc-5* increased the intensity and vesicle size of endosomes enriched for RFP::RAB-5, which includes early endosomes (Figure 2, A–D; see also Figure 1F). RFP::RAB-10 is normally found throughout the cytoplasm. Depletion of *wshc-5* does not significantly change the intensity or size of endosomes enriched for RFP::RAB-10 (Figure 2, A–D). Loss of *wshc-5* increased the intensity and number of RFP::RAB-7 enriched endosomes, measured along the basolateral region, and did not affect vesicle number or cytoplasmic signal (Figure 2, A–E). RFP::RME-1 is normally enriched in basolateral regions. Loss of *wshc-5* resulted in RFP::RME-1-positive recycling endosomes with decreased intensity and decreased vesicle numbers, measured along the basolateral regions, and increased cytoplasmic signal. GFP::RAB-11 is normally highly enriched at apical regions. Depleting *wshc-5* resulted in reduced overall signal, at puncta and in the cytoplasm, most visible at apical puncta along the lumen (2A, white arrows, 2B, apical intensity quantification, 2E). Finally, the Golgi protein AMAN-2::GFP is found in small bright puncta, the Golgi ministacks, throughout the intestine. Depletion of *wshc-5* resulted in increased intensity of AMAN-2::GFP everywhere, and the number of puncta was slightly increased. Collectively, these data suggest an overall shift in endosomal organization, with increased intensity of proteins enriched at early endosomes (RFP::RAB-5), late endosomes (RFP::RAB-7), and the Golgi (GFP::AMAN-2), at the expense of proteins enriched at recycling endosomes (RFP::RME-1, RFP::RAB-10, and GFP::RAB-11; Figure 2, B–E).

Some cargoes travel from early endosomes, with the help of RAB-10-enriched endosomes, to endosomes enriched for RME-1 for further sorting (Chen *et al.*, 2006). These findings could indicate a requirement for WASH at early endosomes for the sorting of cargos normally destined for recycling endosomes. These findings thus encouraged us to investigate where WASH is enriched in the endosomes of the intestine.

### WASH is enriched at some endosomal organelles

**FIGURE 3:**
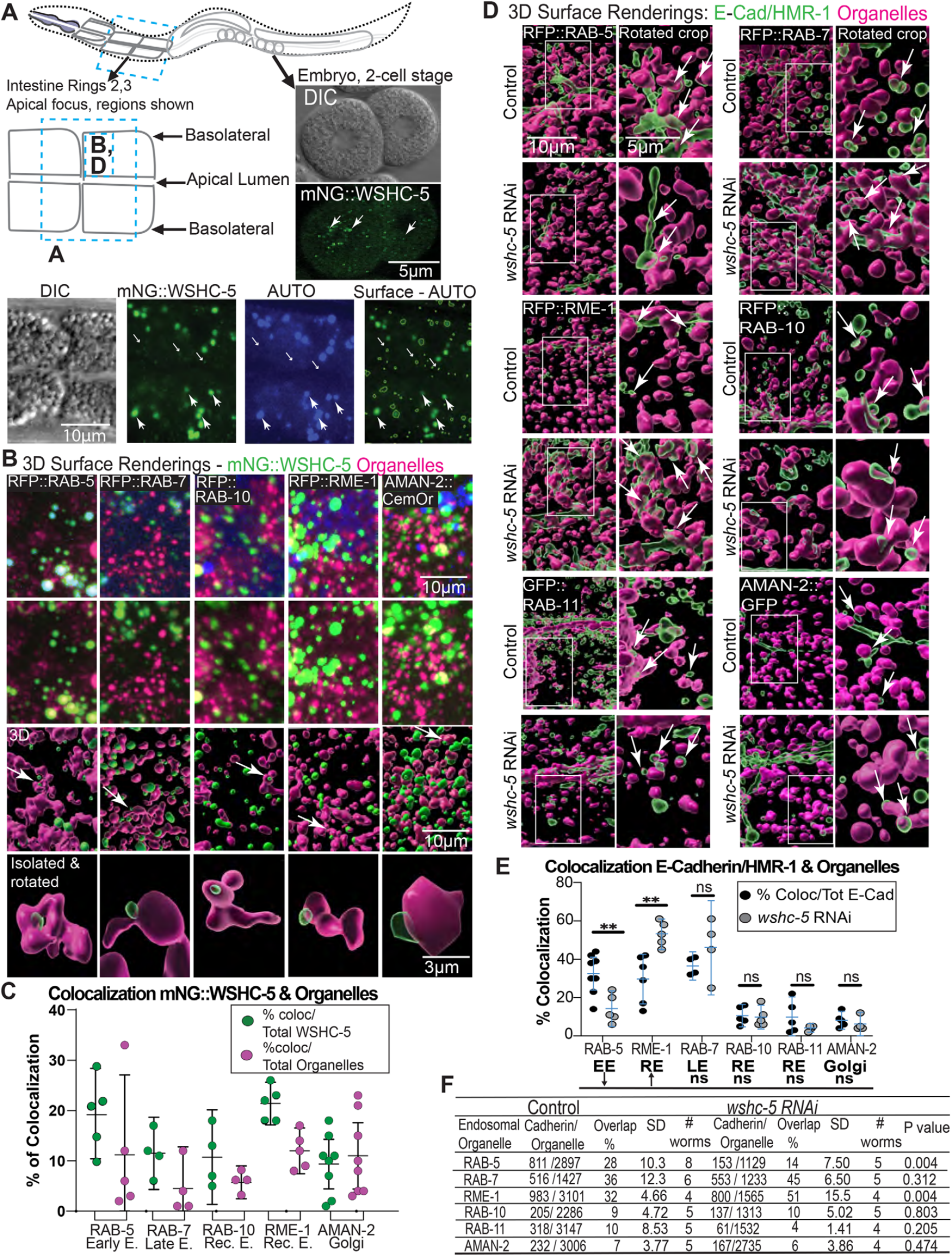
WASH is enriched at recycling endosomes and supports E-Cadherin transport through recycling endosomes. (A) Cartoon worm highlights intestinal region, Rings 2/3, and germline. The expression of mNG::WSCH-5 is shown in a two-cell embryo (top), and in an L4 intestine (below). The boxes with dotted blue lines indicate which regions of the intestine are shown in different parts of the Figure: the smaller box indicates the region shown in 3B and D, while the larger box indicates the region shown in 3A. The *C. elegans* intestine is rich in autofluorescent lysosomal structures which can affect all channels, most visible in the blue channel, labeled here “AUTO” (Autofluorescence). Intestinal autofluorescence (AUTO, blue channel) was not included in the thresholded green channel surface rendering (right panel, circles only around accepted puncta) to better show mNG::WSHC-5 intestinal enrichment. Small downward arrows indicate mNG::WSHC-5 puncta. Large upward arrows indicate large autofluorescent gut granules. (B) *Top row:* Representative raw data 3D projections of Ring 2/3 region of intestine, prepared in Fiji, showing Maximum Projections, with mNG::WSCH-5 in green, autofluorescence in blue and endosomal organelles and the Golgi (labeled “Organelles”) in magenta. Second row: the same images without the blue channel to show that larger green signals overlap autofluorescence. *Third Row:* IMARIS 3D Surface renderings of intestine Ring 2/3 region. The autofluorescence/blue signal was rendered in IMARIS and subtracted from the other channels rendered in IMARIS, magenta and green, before analyzing colocalization. *Bottom row:* Close up of subset of puncta, isolated from neighbors and rotated to illustrate colocalization. The green surfaces are shown partially transparent to better show the overlap. (C) Colocalization of mNG::WSHC-5 (green) and endosomal organelles (magenta). Green dots report % endosomal organelles colocalizing with mNG::WSHC-5 out of total mNG::WSHC-5. Magenta dots report % mNG::WSHC-5 colocalizing with endosomal organelles out of total endosomes. Each dot is the average percent per worm, based on hundreds of measurements done in IMARIS (see Methods and Table in Fig. S1A). Each dot is one worm, *N* = at least 4 worms. (D) Controls and animals depleted of WSHC-5 for 36 hours, are shown, apical focus at Rings 2/3, to show overlap of the surfaces in the green (E-Cadherin/HMR-1) and magenta (endosomal organelles) channels. Arrows point to colocalization regions. White box = area selected for close up to the right, slightly rotated, to show overlap. See also Fig. S1B for additional Close ups of regions of overlap, to better show colocalization from different angles. (E) Colocalization was measured using IMARIS software as in 3B,C. The average for each animal was plotted. Each dot is the average of hundreds of measurements per animal, shown in Table 3F. (F) Table in support of Fig. 3E: Total numbers, after autofluorescence subtraction, of puncta overlap between HMR-1::GFP or HMR-1::mKate2 and the endosomal organelle markers in controls and animals depleted of *wshc-5*, out of total E-Cadherin puncta. Overlap % of the puncta in red and green channels was calculated using IMARIS (see Methods). The % overlap between animals for each condition, based on total number of puncta, were analyzed in Graph Pad Prism, Grouped Analysis, Row statistics and multiple unpaired *t* tests, to generate the SD (Standard Deviation), *P* values and 95% Confidence Intervals. # worms matches # of dots in Fig. 3E.

To determine WASH subcellular enrichment in intestinal epithelia, we used endogenously tagged mNG::WSHC-5, previously localized to puncta in embryos (Zhang *et al.*, 2025; Figure 3A). In adult intestines, we noted mNG::WSHC-5 also enriched at puncta (Figure 3A). Since intestines have autofluorescence produced by the lysosomal gut granules (Morris *et al.*, 2018), we subtracted the autofluorescence signal (blue channel) before analysis (Figure 3A; *Materials and Methods* section).

To test if the WSHC-5 puncta are enriched at specific endosomes or the Golgi, we crossed mNG::WSHC-5 into marker strains tagged with RFP or CemOrange2 (Thomas *et al.*, 2019; Cordova-Burgos *et al.*, 2023). 3D projections of the raw data suggested partial overlap (Figure 3B). Using simultaneous capture imaging, IMARIS 3D rendering, and machine learning approaches to distinguish signals from autofluorescence, we measured colocalization based on overlap volume ratios and nearest neighbor analysis. WSHC-5 was most enriched at RME-1-positive recycling endosomes (21%), followed by early endosomes (19%), RAB-7-positive late endosomes (13%), RAB-10-positive recycling endosomes (11%), and at similar levels at the Golgi (9%) (Figure 3C, green dots, overlap out of total WSHC-5 puncta, see also Supplemental Figure S1). Thus, a significant amount of WASH is enriched at early endosomes and recycling endosomes, which supports a role in transport.

We next examined which endosomes are enriched for E-Cadherin accumulation at steady state in the adult intestinal endothelia by crossing endogenously tagged E-Cadherin, either HMR-1::mKate2 or HMR-1::GFP, into the endosomal strains used for Figure 3, B and C. Using a similar approach as in Figure 3C, we quantified E-Cadherin/HMR-1::GFP enrichment at different endosomal organelles. E-Cadherin was found enriched at 28% of RFP::RAB-5 early endosomes, 36% of RFP::RAB-7 late endosomes and 32% of RFP::RME-1 recycling endosomes, 9% of RFP:: RAB-10 endosomes, 10% of RAB-11::GFP endosomes, and 7% of AMAN-2::GFP Golgi (Figure 3, D and E, dots represent overlap out of total E-Cadherin puncta).

Depleting WASH with *wshc-5* RNAi for 36 h resulted in reduced levels of E-Cadherin overall, and a shift in the distribution of E-Cadherin. E-Cadherin enrichment at RFP::RAB-5 early endosomes dropped significantly from 28%–14%, while RAB-7::RFP late endosomes increased from 36%–45%, but it was not significant (Figure 3, D, E, and F). There was significantly increased E-Cadherin enrichment at RFP::RME-1 recycling endosomes, from 32%–51%. This suggests that E-Cadherin transport from the early endosome and recycling endosomes requires the WASH complex. With reduced WASH complex, E-Cadherin sorting changed, and more accumulated at RME-1-positive recycling endosomes. Decreased E-Cadherin levels seen with depleted WASH are likely due to increased lysosomal degradation, since we showed that blocking lysosomal degradation in animals depleted of WASH, using the *cup-5* mutation, partially restored the levels of E-Cadherin/HMR-1 (Figure 1, D–G). The increased E-Cadherin accumulation seen with depleted WASH at RME-1-positive endosomes was surprising since those endosomes also showed reduced intensity and number (Figure 2).

### *C. elegans* possesses a complete pentameric WASH complex

**FIGURE 4:**
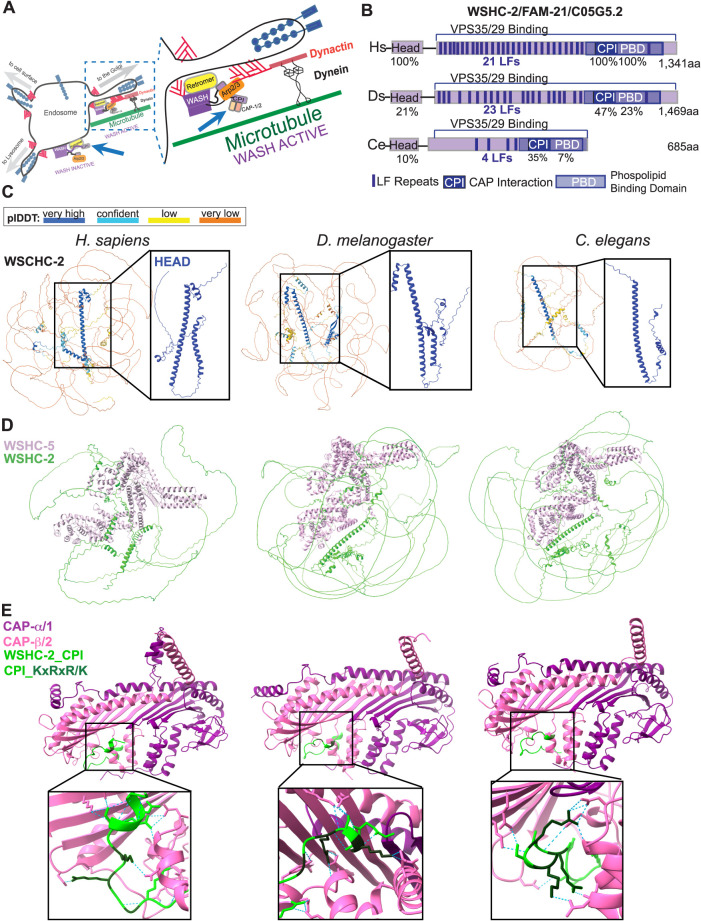
Structural evidence *C. elegans* C05G5.2 is a WSHC-2/FAM-21 ortholog. (A) Cartoon from Figure 1B with enlarged area to highlight roles of WSHC-2/FAM21, the WASH component whose long C-terminal tail binds Retromer (blue arrow on left) and also removes CAPα/1-CAPβ/2 from Dynactin during WASH activation (blue arrow on right, WSHC-2 CPI domain bound to CAP proteins). (B) The WSHC-2/FAM21 ortholog of *C. elegans*, C05H5.2, is divergent at the amino acid level. Domains of *Caenorhabditis elegans* (Ce) C05G5.2 were aligned to WSHC-2/FAM-21 in *Homo sapiens* (*Hs*) and *Drosophila melanogaster* (Ds). Key domains, including the Head domain, LF repeats (dark purple), cap protein interaction domain (CPI), and phospholipid binding domain (PBD), are labeled with conserved identity listed. (C) AlphaFold predictions for the structures of WSHC-2 in *H. sapiens*, *D. melanogaster,* and *C. elegans.* The pIDDT Key indicates confidence levels of AlphaFold's structure prediction. WSHC-2 is predicted to have large unstructured regions (orange regions in all three structures). The boxes show the Head domain enlarged to the right. AlphaFold predicts that the Head domain in all three proteins forms a similar long alpha helix plus additional smaller alpha helices (blue, very high confidence). (D) ColabFold predictions of WSHC-2 (green) assembled with WSHC-5 (magenta) in *H.* sapiens, *D. melanogaster*, and *C. elegans*. (E) ColabFold prediction of WSHC-2 CPI domain (green) wrapping around the stalk region of the two capping proteins (CAP-α, purple, CAP-β, pink). Boxes show enlarged stalk/CPI binding, with CPI in green, essential KxRxR/K residues in darker green, and hydrogen bonds in dotted light blue. Structural data for this paper were retrieved from the Alliance of Genome Resources (Bult and Sternberg, 2023) https://www.alliancegenome.org, May 2026, release version 9.0.0.

To further address the role of *C. elegans* WASH in the transport of polarized cargos, we needed to identify the expected five components of *C. elegans* WASH complex (Figure 1A). One problem was the apparent lack of a WSHC-2/FAM21 ortholog. WSHC-2/FAM21 function is central to WASH complex function in other species, since its long C-terminal tail connects WASH to two types of partner molecules at endosomes: Retromer components (VPS-29, VPS-35, and VPS-26) and the Capping Proteins (CapZα/CAP-1 and CapZβ/CAP-2; Figure 4A). Retromer promotes retrograde transport of proteins, while Capping Proteins are displaced when WASH binds to the Dynactin–Dynein complex during WASH activation (Figure 4A; Seaman *et al.*, 1998; Hierro *et al.*, 2007; Fokin *et al.*, 2021; Romano-Moreno *et al.*, 2024). However, others have noted that WSHC-2/FAM21 can be highly divergent. For example, Velle and colleagues showed that using a mutual-best-BLAST-hit approach, *Drosophila* did not appear to have a FAM21 ortholog (Velle *et al.*, 2024). The Billadeu lab suggested that CO5G5.2 may be the WSHC-2/FAM21 ortholog of *C. elegans* (Wang *et al.*, 2018).

Aligning the highly divergent protein, CO5G5.2, with human and *Drosophila* WSHC-2/FAM21 revealed a likely reason this WASH component was initially missed (Figure 4B). The proposed *C. elegans* WSHC-2/FAM21 protein is about half the length of orthologs in other organisms. In addition, CO5G5.2 contains highly divergent WSHC-2/FAM21 motifs, as defined in Jia *et al.*, 2010. Compared with human WSHC-2/FAM21, the N-terminal “head” domain (Jia *et al.*, 2012), predicted to bind WSHC-1/DDL-2, showed only 10% identity over 220 residues, the Phospholipid binding domain that binds Retromer, showed 7% identity over 404 residues, and the Cap Protein Interacting (CPI) motif, which binds to CAPZ proteins, showed 35% identity over 17 residues. Human WSHC-2/FAM21 has 21 LF (Leu-Phe) motifs, which connect WSHC-2 to Retromer components VPS35 and VPS29 (Jia *et al.,* 2012; Romano-Moreno *et al.*, 2024). *Drosophila* has 23, but *C. elegans* has only 4 LF motifs (Figure 4B).

Structural analysis suggests *C. elegans* WSHC-2 is the likely WSHC-2 ortholog, despite limited conservation with its human and *Drosophila* orthologs. AlphaFold (Jumper *et al.*, 2021) predicts that the major motifs are more conserved than the amino acid alignments would suggest. For example, the Head domain, only 10% identical, contains a characteristic long alpha helix that is predicted with “very high” confidence (Figure 4, C and D). Alphafold further predicted that the 4 LFs are similarly part of a long unstructured region, like in humans and *Drosophila* (Figure 4D). Structural work on human FAM21 suggested that while WSHC-2/FAM21 has 21 LF repeats, only a few bind to retromer. Only FAM21 repeats 1–4 and 20, 21 are important for binding to retromer, and only 20 and 21 bind singly and strongly (Romano-Moreno *et al*., 2024). Thus, it is possible that a WSHC-2/FAM21 ortholog with only 4 LF repeats could carry out WSHC-2/FAM21 retromer binding.

We used ColabFold alignment (Mirdita *et al.*, 2022) to predict how the *C. elegans* WSCH-2 may interact with expected protein partners. ColabFold alignment of the WSHC-2 and WSHC-5 orthologs of *C. elegan*s, humans, and *Drosophila* suggested the two WASH components can organize into similar assemblies in all three organisms (Figure 4D). To examine if the 6/17 conserved residues in the CPI consensus motif (Jia *et al.,* 2010; Hernandez-Valladares *et al.*, 2010) may permit *C. elegans* WSHC-2 to bind to Capping Proteins, CapZA/α/CAP-1 and CapZB/β/CAP-2 (Figure 4, A and B), we noted that the three residues essential for CP binding (KxRxK/R) are conserved or similar (KxRxR) in WSHC-2/C05G5.2 (Figure 4E). The two Capping Proteins assemble into a mushroom shape, and the CPI motif of WSHC-2 binds the beta subunit at the mushroom stalk, using the essential (KxRxK/R) residues (Hernandez-Valladares *et al.,* 2010). ColabFold assembly of the two capping proteins with the cap protein interacting (CPI) motif of the WSHC-2 orthologs from humans, *Drosophila*, and *C. elegans* predicted that the CPIs in all three species can similarly bind to the stalk region, making multiple hydrogen bonds between the KxRxK/R and Capβ/CAP-2 (dotted blue line in Figure 4E). Thus, the highly diverged WSHC-2/FAM21 ortholog CO5G5.2 is likely a component of the *C. elegans* WASH complex, and we refer to it hereafter as WSHC-2.

### The proposed WSHC-2/FAM21ortholog has a role in retromer transport

**FIGURE 5:**
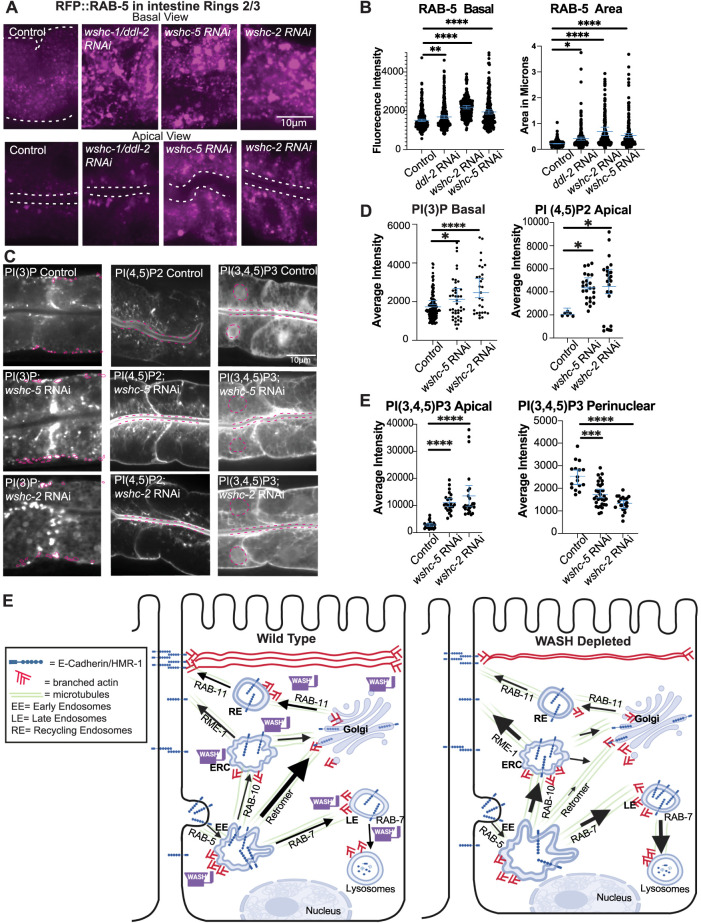
WASH components, including *C. elegans* WSHC-2, affect retromer transport and regulate HMR-1/E-E-Cadherin transport. (A) Loss of WSHC-2/FAM21 is compared with loss of WASH components WSHC-5/Strumpellin and WSHC-1/DDL-2, using RNAi for 36 h, at RFP::RAB-5 enriched early endosomes. Top row: Basal view, dotted line shows outline of the intestine; Bottom row: Apical view, dotted lines show apical lumen. (B) Quantification of vesicle intensity and vesicle area of RFP::RAB-5 endosomes, measured using the Freehand tool in Fiji to circle vesicles, as in Figure 2. (C) Effect of WSHC-5/Strumpellin and WSHC-2/FAM21depletion, by RNAi for 48 h, on phospholipid levels and distribution in the intestinal cells of *C. elegans*. The distribution of membrane lipids PI(3)P; PI(4,5)P2; and PI(3,4,5)P3 is shown from the Apical view, which allows measurement of apical, lateral, and basal regions. (D) Fluorescence intensity was measured in FIJI using the line tool for PI(3)P basal signal, PI3,4,5)P3 apical or PI(4,5)P2 apical signal. The freehand tool was used to measure PI(3,4,5)P3 perinuclear signal. (E) Model summarizing the observed changes in the sorting of E-Cadherin cargo in controls and in animals depleted of WASH components. Loss of WASH components led to enlarged early endosomes, and increased transport of E-Cadherin/HMR-1::GFP to RAB-7-positive late endosomes, and to RME-1/EHD-1-positive recycling endosomes. Levels of E-Cadherin dropped overall, including at the apical junction, which may explain the loss of apical F-actin. Figure 5D, except for some labeling, was created in BioRender. Irizarry-Barreto, P. (2026) https://BioRender.com/6yjq79q

WSHC-2/FAM21 and the WASH complex regulate Retromer-dependent sorting (Gomez and Billadeau, 2009). If the *C. elegans* WSHC-2 ortholog works with WASH to support retromer transport from early endosomes to the Golgi, loss of WSHC-2 was expected to alter the early endosomes (Figures 2 and 3). In support of a Retromer function, loss of WSHC-2, similar to loss of WSHC-5 or WSHC-1/DDL-2 using RNAi depletion, led to increased intensity and size of early endosomes enriched for RFP::RAB-5 (Figure 5, A and B), and as was shown for RAB-5 early endosome morphology in the absence of retromer components (Bai and Grant, 2015).

### *C. elegans* WASH regulates membrane enrichment of phospholipids

Changes in endosome organization could result in changes in the phosphatidylinositol phospholipid (PIP) composition that establishes distinct cellular membranes. To further investigate the consequences of *WSHC-2* depletion, we tested the effects of WSHC-2 and other WASH component depletion on phospholipids. RAB-5 recruits PI3 kinase to produce and enrich phosphatidylinositol 3-monophosphate (PI(3)P) on early endosomes. Depletion of WASH components *wshc-5* or *wshc-2* for 48 h significantly increased PI(3)P enrichment and intensity. This result supports the idea that WSHC-2 behaves similarly to other WASH components in regulating PI(3)P enrichment. Two other phospholipids, Phosphatidylinositol-4,5-bisphosphate (PI(4,5)P2) and phosphatidylinositol-3,4,5-triphosphate (PI(3,4,5)P3), are normally enriched at the plasma membrane (Shi *et al*., 2012). RNAi depletion of *wshc-5* or *wshc-2* increased plasma membrane enrichment of PI(4,5)P2 and PI(3,4,5)P3 (Figure 5, C and D). For PI(3,4,5)P3, we noted a significant drop in the perinuclear accumulation when all WASH components, including WSHC-2, were depleted. While the molecular mechanisms that result in these phospholipid changes are not yet clear, these data demonstrate that WSHC-2 loss phenocopies loss of other WASH components.

### Model for how WASH supports polarized transport

We propose that *C. elegans* has a diverged yet structurally similar WSHC-2 ortholog that contributes to WASH function (Figures 4 and 5). WASH in *C. elegans* appears to support retromer transport (Figure 5A), as depleting WASH components leads to altered early endosomes and recycling endosomes, and increased intensity and number for markers of late endosomes (Figure 2). One important epithelial cargo, E-Cadherin/HMR-1, depends on WASH for normal levels, in part due to a strong requirement for WASH at RAB-5 and RME-1/EHD1 enriched endosomes (Figures 2 and 3, B and C). Loss of WASH components also affects E-Cadherin sorting to recycling endosomes (Figure 3, D–F). Of note, loss of WASH had a distinct role on E-Cadherin transport, as compared with the effects of depleting WAVE, which did not affect early endosomes, but strongly affected RAB-11-positive recycling endosomes (Cordova-Burgos *et al.*, 2023). These findings support the model (Figure 5E) that each Arp2/3 NPF has a distinct role in endocytosis and highlight the need to identify partner molecules that bring the distinct NPFs, WASH, WAVE, and WASP, to distinct endosomal organelles to support sorting that then supports polarized distribution of cargos.

## MATERIALS AND METHODS

Request a protocol through *Bio-protocol*

### *C. elegans* strains built for this paper

The following strains were built by crossing pre-existing strains, referenced below under “Strains used in this paper.” OX237 *mNG::wshc-5; gfp; tagRFP::rab-5;* OX1082 *mNG::wshc-5; tagRFP::rab-7;* OX1080 *mNG::wshc-5; tagRFP rab-10; OX1084 mNG::wshc-5; tagRFP::rme-1;* OX1083 m *mNG::wshc-5; aman-2::CemOr;* OX1081 *mNG::wshc-5; pGlo-1::LAmCh.*

### Strains used in this paper

Some of these strains were endogenously tagged using CRISPR: LP902 *mNG::wshc-5* (Zhang *et al.*, 2025); LP172 *hmr-1::gfp*. h*mr-1::mKate-2 (**Marston et al., 2016**).* The rest are integrated multi-copy arrays.

OX983 *hmr-1::mKate-2; aman2::gfp(**Cordova-Burgos et al., 2023**);* OX902 *hmr-1::gfp; tagRFP::rme-1(**Cordova-Burgos et al., 2023**);* OX810 *hmr-1::gfp; tagRFP::rab-5 (**Cordova-Burgos et al., 2023**);* OX806 *hmr-1::gfp; tagRFP::rab-7(**Cordova-Burgos et al., 2023**);* OX901 *hmr-1::gfp; tagRFP rab-10 (**Cordova-Burgos et al., 2023**)*; OX971 *hmr-1::mKate2*; *gfp::rab-11 (**Cordova-Burgos et al., 2023**);* OX1019 *pjIs17 [nhx-2p::aman-2::CemOrange2] (**Cordova-Burgos et al., 2023**)*; LP172 *hmr-1::gfp* (Marston *et al.*, 2016); *tagRFP::rab-5* (Gleason *et al.*, 2016); *tagRFP::rab-10* (Gleason *et al.*, 2016); *tagRFP::rab-7* (Gleason *et al.*, 2016); *tagRFP::rme-1* (Gleason *et al.*, 2016); RT311 *vha-6p::gfp::rab-11* (Chen *et al.*, 2006); OX966 *pGlo-1::TAG::RFP*; pRF4 *rol-6(su1006)* (Cordova-Burgos *et al.*, 2021).

### RNAi experiments

All RNAi bacterial strains used in this study were administered by the feeding protocol as in (Sasidharan *et al.*, 2018). RNAi feeding experiments were done at 23°C unless otherwise mentioned. Worms were synchronized and transferred onto a seeded plate containing RNAi-expressing bacteria. To monitor the effectiveness of the RNAi, we used two methods: (1) We counted the percent dead embryos, which after two days is expected at >90% for *gex-3* and at least 10% for *wshc-5;* (2) We monitored post-embryonic silencing of a mNG-tagged strain in the intestine, LP902 *mNG::wshc-5*. Each Figure reports if RNAi treatments were done for 24, 36, or 48 h. For example, Phospholipid strains were treated for 48 h while organelle and E-Cadherin/HMR-1 strains were treated for 36 h, so the Controls were monitored at the same time points.

### Live imaging

Imaging was done in a temperature-controlled room set to 23°C on a Laser Spinning Disk Confocal Microscope with a Yokogawa scan head, on a Zeiss AxioImager Z1 Microscope using the Plan-Apo 63X/1.4NA oil lens. Imaging for colocalization studies used Visiview software and two identical Hamantsu CMOS Cameras to simultaneously capture two fluorophores. Each Figure explains imaging conditions, and only experiments done under the same conditions were compared. Images were analyzed using ImageJ or IMARIS, as explained in the Figure Legends. Controls and mutants were imaged within 3 d of each other with the same imaging conditions. All measurements were performed on raw data using ImageJ and/or IMARIS. Background intensity was subtracted by measuring the average intensity in the same focal plane, near the animal.

### Microscopy of L4s and adults

Young adult stage animals (one day after L4 stage) and L4 larvae were placed on 10% agar pads in M9 solution and immobilized using 2 µl of Levamizole(100 µM) salts and covered with 1.5 um coverslips. Images were taken within 15 min of making the pads. Imaging was done on a Zeiss AxioImager Z1 with a Yokogawa CSUX1-5000 spinning disk, using the Plan Apo 63X/1.4NA Oil lens.

### Quantitation of immunofluorescence

Quantitation of live fluorescence was performed using the line selection and the dynamic profile function of ImageJ to measure fluorescence along lines of equal length, for example, the apical intestine or lateral regions of the intestinal cells. Some puncta were measured using the Freehand Tool to detect size changes. For all experiments shown, the images were captured at the same exposure settings for wild type and mutants. All quantification was done on the raw images. The figure legends indicate when images were enhanced for contrast, and the same enhancement was applied to a mosaic of the related images for that experiment. Each measurement was taken following the subtraction of background fluorescence.

### Rationale for the colocalization method used

In our organism, the presence of the intestinal granules creates large regions of autofluorescence (seen in the blue channel). We acquired the IMARIS system, which creates surface renderings of the puncta, and allowed us to remove autofluorescence by subtracting signal from the blue channel that bled into other channels, using different thresholding and machine learning protocols. We compared intensity-based and pixel-classification-based surface renderings and chose intensity-based rendering.

### Subtraction of autofluorescence and colocalization calculations

To calculate colocalization of red and green channels, we used the algorithm object-to-object to generate 3D surfaces rendered based on signal intensity in the red, green, and blue channels. We manually adjusted the intensity thresholds to include the expected puncta, and used the Classification tool that includes a machine learning (ML) component to train the software to recognize red and green signals that overlapped with the blue channel, and then removed red and green surfaces that overlapped with the blue surfaces. The red and green surfaces were then analyzed for colocalization based on object-to-object statistics, specifically looking at the overlap volume ratio to surface and the shortest distance to surface (surface edge to surface edge overlap). We report the % overlap for each channel out of all visible objects in that channel (Figures 2 and 5). Due to the autofluorescence subtraction, the colocalization measured here may undercount how much mNG::WSHC-5 or E-Cadherin/HMR-1 is enriched at endosomes.

### Statistical analysis

All statistical analyses were performed using GraphPad Prism 8. For most grouped data, statistical significance was established by performing a one-way Analysis of Variance (ANOVA), the Brown-Forsythe and Welch ANOVA, followed by a Dunnett's multiple comparisons T3 post-test. For Figure 3F, Supplemental Figure S1A, we used Prism, Grouped Analysis, Row statistics, and multiple unpaired *t* test, which generated SD, *P* values, and 95% confidence intervals. Two variable groups were compared with the following parameters: (1) Experimental design: unpaired; (2) Distribution assumption: Normal (Gaussian); and (3) No assumption about consistent SDs. For ungrouped data in Figure 2, B–E, an unpaired *t* test, the unequal variance (Welch) *t* test, was used for each pair. Error bars show 95% confidence intervals. Asterisks (*) denote *p* values * = *p* < 0.05, ** = *p* < 0.01, *** = *p* < 0.001, **** = *p* < 0.0001.

### AlphaFold and ColabFold assemblies

To assemble AlphaFold and ColabFold structural predictions, protein sequences from Alliance (Bult and Sternberg, 2023) were imported into the servers. In ColabFold, the *model_type* selected under “Advanced Settings” was *alphafold2_multimer_v3* as stated in Mirdita *et al.* 2022. The resulting *.pdb* file was imported into UCSF ChimeraX (Pettersen *et al*., 2021) to add colors and show hydrogen bonds.

## Supporting information





## Abbreviations used:

C. elegans: Caenorhabditis elegans
cup-: Coelomocyte UPtake-defective
E-Cadherin: Epithelial Cadherin
ER: endoplasmic reticulum
F-actin: filamentous actin
gex-: Gut on the Exterior
HMR-: HaMmeRhead embryonic lethal
NPFs: nucleation-promoting factors
RAB: Ras-related in brain
RME: Receptor Mediated Endocytosis
WASP: Wiskott-Aldrich syndrome protein
WASH: WASP and Scar homolog
WSHC-n: WASH Complex subunit
WAVE: WASP-family verprolin-homologous protein.
