# Supplementary material for: The C. elegans WASH complex supports epithelial polarity by promoting endosomal sorting of E-Cadherin
Source: Mol Biol Cell. 2026 Jun 11;37(8):br24. doi: 10.1091/mbc.E25-12-0612 (PMC13428335; doi:10.1091/mbc.E25-12-0612)
Supplement: Supplementary file 1 [file mbc-37-br24-s001.pdf]

Supplemental Materials

Molecular Biology of the Cell

Irizarry-Barreto *et al*.

A

% coloc/ Total WSHC-5			% coloc/ Total Organelles		
Endosomal Organelle	Organelles/ Tot. WSHC-5	Overlap %	WSHC-5/ Tot. Organelles	Overlap %	# worms
RAB-5	517/2541	19	464/4374	11	5
RAB-7	383/3884	13	168/4176	5	4
RME-1	1360/6673	21	1029/8645	10	4
RAB-10	269/2954	11	214/3902	6	4
AMAN-2	458/5451	9	483/4746	6	8

B

3D Surface Renderings -
E-Cadherin/HMR-1 & Endosomes

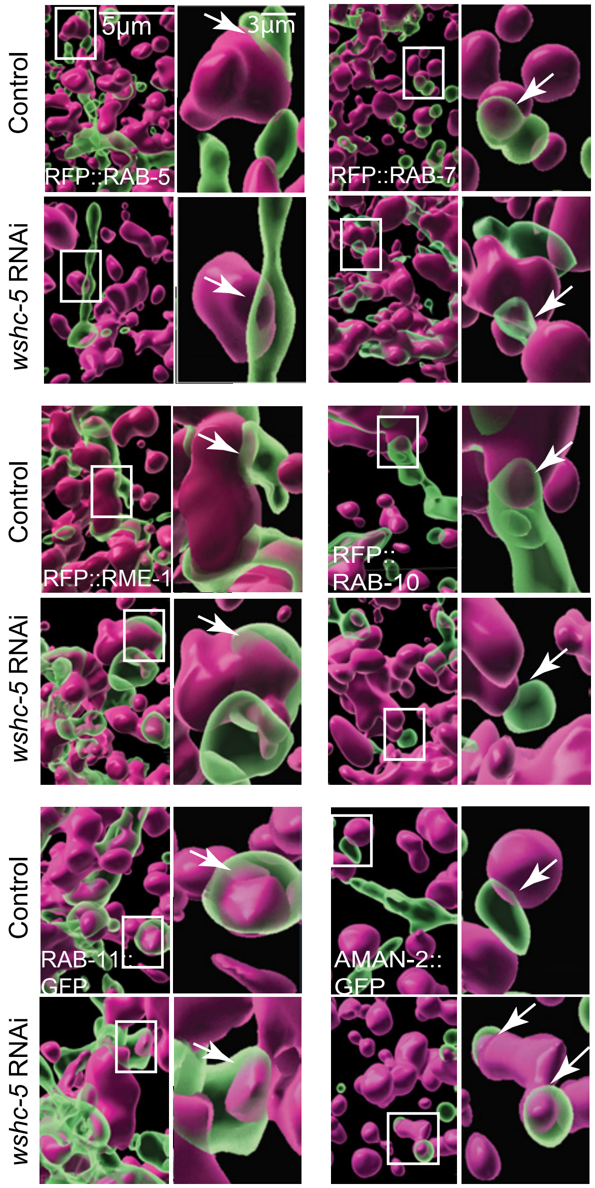

Fig. S1. Additional IMARIS surface renderings and Table related to images shown in Fig. 3C and 3D.
(A) Table 1 supports Fig. 3C: Total numbers, after autofluorescence subtraction, of puncta overlap between mNG::WSHC-5 and the endosomal organelle markers. Overlap % of the puncta in red and green channels was calculated using IMARIS (see Methods). The % overlap between mNG::WSHC-5 and endosomal organelles, based on total number of puncta, were analyzed in Graph Pad Prism, Grouped Analysis, Row statistics and multiple unpaired t-tests, to generate the SD (Standard Deviation), P values and 95% Confidence Intervals used in 3C.
(B) Additional samples and crops of the imaging shown in Figure 3D, to show more instances of E-Cadherin/HMR-1 colocalization with endosomal organelles. 3D rendering of endosomes and the Golgi overlapping with E-Cahedrin/HMR-1 (left) and close up (right) of E-Cadherin and endosomal or Golgi overlap after subtraction of autofluorescence.
