# Supplementary material for: The C. elegans WASH complex supports epithelial polarity by promoting endosomal sorting of E-Cadherin
Source: Mol Biol Cell. 2026 Jun 11;37(8):br24. doi: 10.1091/mbc.E25-12-0612 (PMC13428335; doi:10.1091/mbc.E25-12-0612)
Supplement: Supplementary file 2 [file reviewer_reportE25-12-0612.pdf]

The C. elegans WASH complex supports epithelial polarity by promoting endosomal sorting of E-Cadherin

Martha Soto, Patricia Irrizarry-Barreto, Jennifer Smolyn, Victoria Brown, and Bhagavathy Ramamurthy

Corresponding author(s): Martha Soto, Rutgers, The State University of New Jersey

Review Timeline:

Submission Date:	2025-12-23
Editorial Decision:	2026-02-20
Revision Received:	2026-05-22
Accepted:	2026-06-02

Editor-in-Chief: John Aitchison

Transaction Report:

(Note: With the exception of the correction of typographical or spelling errors that could be a source of ambiguity, letters and reports are not edited. The original formatting of letters and referee reports may not be reflected in this compilation.)

RE: Manuscript #E25-12-0612
TITLE: The C. elegans WASH complex supports epithelial polarity by promoting endosomal sorting of Cadherin

Dear Dr. Soto:

I apologize that the review process has taken so long. We have now received reviews from two experts in the field. Both reviewers were positive about the potential significance of the work, but identified problems that preclude publication in Molecular Biology of the Cell in its current form. Most significantly, both reviewers share concerns about the claim that C05G5.2 is the FAM21 ortholog and provide suggestions for work that would enhance that claim. Reviewer 1 also has concerns about sample sizes and statistical analyses that will need to be addressed. There are also concerns about data from a master's thesis that is not readily accessible, and conclusions that are not strictly supported by the data. Additionally, the MBoC submission requirements have not been met with regard to specifying sample size. A revised manuscript would need to address these and other concerns.

Sincerely,

Joshua Kelley
Monitoring Editor
Molecular Biology of the Cell

Dear Dr. Soto,

The review of your manuscript, referenced above, is now complete. The Monitoring Editor has decided that your manuscript is not acceptable for publication at this time, but may be deemed acceptable after specific revisions are made, as described in the Monitoring Editor's decision letter above and the reviewer comments below.

A reminder: Please do not contact the Monitoring Editor directly regarding your manuscript. If you have any questions regarding the review process or the decision, please contact the MBoC Editorial Office (mboc@ascb.org).

When submitting your revision include a rebuttal letter that details, point-by-point, how the Monitoring Editor's and reviewers' comments have been addressed. (The file type for this letter must be "rebuttal letter"; do not include your response to the Monitoring Editor and reviewers in a "cover letter.") Please bear in mind that your rebuttal letter will be published with your paper if it is accepted, unless you have opted out of publishing the review history.

Authors are allowed 90 days to submit a revision. If this time period is inadequate, please contact us at mboc@ascb.org.

Revised manuscripts are assigned to the original Monitoring Editor whenever possible. However, special circumstances may preclude this. Also, revised manuscripts are often sent out for re-review, usually to the original reviewers when possible. The Monitoring Editor may solicit additional reviews if it is deemed necessary to render a completely informed decision.

In preparing your revised manuscript, please follow the instruction in the Information for Authors (www.molbiolcell.org/info-for-authors). In particular, to prepare for the possible acceptance of your revised manuscript, submit final, publication-quality figures with your revision as described.

To submit the rebuttal letter, revised manuscript, and figures, use this link: Link Not Available

MBoC offers the option to publish your work with immediate open access. Open access can increase the discoverability and usability of your research. If you would like to publish your paper with immediate open access but did not select this option during initial submission, please contact the editorial office at mbc@ascb.org.

Authors of Articles and Brief Communications are also encouraged to create a short video abstract to accompany their article when it is published. These video abstracts, known as Science Sketches, are up to 2 minutes long and will be published on YouTube and then embedded in the article abstract. Science Sketch Editors on the MBoC Editorial Board will guide you as you prepare your video. Information about how to prepare and submit a video abstract is available at www.molbiolcell.org/science-sketches. Please contact mboc@ascb.org if you are interested in creating a Science Sketch.

Sign up for MBoC's new content alerts here https://tinyurl.com/3bcsyk3e

Thank you for submitting your manuscript to MBoC. We look forward to receiving your revised paper.

Sincerely,

Eric Baker
Journal Production Manager
MBoC Editorial Office
mbc@ascb.org

Reviewer #1 (Remarks to the Author):

The evidence that WASH is required for localization of E-cadherin/HMR-1 to the intestinal junctions in C. elegans adults and the role for WASH in different endosomal compartments is strong and provides insight into the role of another NPF.

I think that this paper has some interesting data that may support the claim that C05G5.2 is the C. elegans FAM21 ortholog. However, given how divergent the protein is from the human and fly FAM21, I think stronger evidence is required to justify this claim. The methods should also include details on how C05G5.2 was identified as a likely FAM21 ortholog, particularly given its divergence.

Sample sizes are often small (sometimes only 3 animals were examined) and exact sample sizes are not often given. Please ensure that exact sample sizes are given for every experiment in the corresponding figure legend.

For the significance statements, I don't think there is sufficient evidence to claim that "WASH branched actin polarizes epithelia using a functional FAM21 ortholog". This statement should be tempered, or additional evidence needs to be provided.

Additional detailed comments are provided in my attached review.

Reviewer #2 (Remarks to the Author):

This manuscript by Irizarry-Barreto et al. presents a solid characterization of the WASH complex in C. elegans, filling an important gap in our understanding of how different NPFs contribute to cargo trafficking. The authors demonstrate that WASH components support Cadherin localization and epithelial polarity, identify the highly divergent C05G5.2 as the likely FAM21 ortholog, and show that WASH functions at early and recycling endosomes to regulate retrograde transport. The work is technically sound with appropriate quantitative analyses, and the phenotypes are clear and statistically significant.
The systematic characterization of WASH complex localization and function in C. elegans represents valuable foundational work for the field. The quantitative imaging approaches are rigorous and well-executed. The identification of distinct roles for WAVE versus WASH in endosomal trafficking provides important mechanistic insights. Statistical analyses are appropriate and the manuscript is clearly written with helpful models and figures.
Major Issues:
1. Two important findings are referenced solely from an unpublished master's thesis (Smolyn, 2020). These data provide important context for the current findings. These data should be included in the supplemental material rather than left inaccessible in a master's thesis.
2. Identification of C05G5.2 as FAM21 is reasonably convincing but could be easily strengthened. The authors make a reasonable case that C05G5.2 is the FAM21/WSHC-2 ortholog based on functional data and limited sequence similarity in key domains (pages 4-5). However, given the low sequence identity (10% for the head domain, 7% for the phospholipid binding domain), additional structural predictions would significantly strengthen this conclusion. I recommend using AlphaFold predictions for C05G5.2 compared to human and Drosophila FAM21 to determine if the predicted folds are more conserved than the sequences. Furthermore, ColabFold analysis to predict conserved protein-protein interactions, particularly with other WASH components, Retromer, and capping proteins. Additionally, once C05G5.2 is established as WSHC-2, the authors should use "WSHC-2" consistently throughout the manuscript rather than waiting until page 6 to adopt this nomenclature. The gene name "C05G5.2" need only appear once upon introduction.
3. In the Methods section, the "C. elegans strains built for this paper" section is inadequate. The authors list strain names but provide no information on construction methods. How were fluorescent tags inserted? (CRISPR/Cas9, MosSCI, other method?) What are the relevant guide RNA sequences and/or primers used?
Minor point:
The autofluorescence subtraction method is not fully described in the methods. What kind of thresholding and machine learning did you use? Is this useful for others, and can it be made available?

RE: Manuscript #E25-12-0612
TITLE: The C. elegans WASH complex supports epithelial polarity by promoting endosomal sorting of E-Cadherin
----

Dear Drs. Kelley and Baker,

We have addressed all of the Reviewers' queries, as can be seen in the point-by-point responses below. In response to their questions and suggestions, we added additional experiments to Figures 1,2, 3 and 4. References to experiments in the Master's Thesis were removed and replaced by new experiments to directly address those points (Revised Figure 1G-J), and an updated link to the Master's Thesis was added, since it is clear people in our field are using data from the Thesis. Figure 4 was revised to address the Reviewers questions about the similarity of *C. elegans* C05G5.2 with human and *Drosophila* WSHC-2/FAM21. New structural alignments using AlphaFold and ColabFold predictions, suggested by one of the Reviewers, strongly support our functional data (in revised Figure 5), further supporting that we have identified the correct WSHC-2/FAM21 ortholog. Sample sizes were added to all Figures and statistics were reviewed and revised.

Please let us know if you need anything further.
best,
Martha Soto

Reviewer #1 (Remarks to the Author):

The evidence that WASH is required for localization of E-cadherin/HMR-1 to the intestinal junctions in C. elegans adults and the role for WASH in different endosomal compartments is strong and provides insight into the role of another NPF.

I think that this paper has some interesting data that may support the claim that C05G5.2 is the C. elegans FAM21 ortholog. However, given how divergent the protein is from the human and fly FAM21, I think stronger evidence is required to justify this claim. The methods should also include details on how C05G5.2 was identified as a likely FAM21 ortholog, particularly given its divergence.
Response: We have added on page 5 that the original connection between C05G5.2 came directly from a 2018 Review written by the Billadeau lab [Wang et al., 2018 doi: 10.1111/tra.12574]. That Review did not explain exactly how they found the similarity. Therefore, we did extensive comparisons, better described now on page 5, and tested knockdown of this FAM21 candidate by RNAi. **In the Revised manuscript, we add**:

1) This citation:
Wang J, Fedoseienko A, Chen B, Burstein E, Jia D, Billadeau DD. Endosomal receptor trafficking: Retromer and beyond. Traffic. 2018 Aug;19(8):578-590. doi: 10.1111/tra.12574. Epub 2018 May 21. PMID: 29667289

2) Revised Figure 4: Alpha Fold alignment, showing that despite low similarity at the amino acid level, the key WSHC-2/FAM21 motifs are highly conserved and are predicted to assemble into similar structures (Revised Fig. 4C).

3) ColabFold predictions, showin*g that WSHC-2 and WSHC-5 association in C. elegans is similar to that in H. sapiens and* D. melanogaster (Revised Fig. 4D), and ColabFold predictions of how the FAM21 CPI binds to the Capping Proteins, again showing that the *C. elegans* protein is predicted to interact similarly, using conserved CPI residues (Fig. 4E).

Sample sizes are often small (sometimes only 3 animals were examined) and exact sample sizes are not often given. Please ensure that exact sample sizes are given for every experiment in the corresponding figure legend.
Response: We thank the Reviewer for pointing his out. We have added exact sample sizes for each experiment in the revised Figure Legends. Additional experiments were done to increase sample sizes, especially for RAB-10 in Figure 2.

For the significance statements, I don't think there is sufficient evidence to claim that "WASH branched actin polarizes epithelia using a functional FAM21 ortholog". This statement should be tempered, or additional evidence needs to be provided.
Response: We have added additional evidence that we have identified a functional FAM21 ortholog, Fig. 4, and have tempered the significance statement, which now reads, *WASH branched actin polarizes epithelia to promote sorting of retrograde cargo, including E-Cadherin.".

The second significance statement now reads, "*We show that *C. elegans* C05G5.2 is a likely FAM21/WSHC-2 ortholog, since it shares structural features with FAM21 orthologs. Loss of WASH components, including the WSHC-2 ortholog, altered retrograde transport and reduced epithelial polarity, while the cargo -E-Cadherin was mis-sorted from early endosomes. "

Additional detailed comments are provided in my attached review. [See after Reviewer #2.]
We respond to these below the Reviewer #2 comments.

Reviewer #2 (Remarks to the Author):

This manuscript by Irizarry-Barreto et al. presents a solid characterization of the WASH complex in C. elegans, filling an important gap in our understanding of how different NPFs contribute to cargo trafficking. The authors demonstrate that WASH components support Cadherin localization and epithelial polarity, identify the highly divergent C05G5.2 as the likely FAM21 ortholog, and show that WASH functions at early and recycling endosomes to regulate retrograde transport. The work is technically sound with appropriate quantitative analyses, and the phenotypes are clear and statistically significant.
The systematic characterization of WASH complex localization and function in C. elegans represents valuable foundational work for the field. The quantitative imaging approaches are rigorous and well-executed. The identification of distinct roles for WAVE versus WASH in endosomal trafficking provides important mechanistic insights. Statistical analyses are appropriate and the manuscript is clearly written with helpful models and figures.
Response: We thank the Reviewer for appreciating the effort that went into this first analysis of the role of the WASH complex in *C. elegans.*

Major Issues:

1. Two important findings are referenced solely from an unpublished master's thesis (Smolyn, 2020). These data provide important context for the current findings. These data should be included in the supplemental material rather than left inaccessible in a master's thesis.
Response: We conducted new experiments and added the data to Figure 1, to show that:

a) we can rescue loss of Cadherin in a WSHC-5 depleted animal by blocking lysogenesis with the *cup-5* mutant (Fig. 1 G,H);
b) Loss of WASH components, just like loss of WAVE components, reduces apical F-actin in the intestine (Fig. 1I,J).

We were going to completely remove the Master's Thesis, except that we keep finding evidence that other scientists are finding it, and using it to guide their experiments. We don't rely on any data from the Master's Thesis, but we mention it exists and added an updated link that should be accessible to all. Smolyn 2020: https://doi.org/doi:10.7282/t3-sgsb-mr58

2. Identification of C05G5.2 as FAM21 is reasonably convincing but could be easily strengthened. The authors make a reasonable case that C05G5.2 is the FAM21/WSHC-2 ortholog based on functional data and limited sequence similarity in key domains (pages 4-5). However, **given the low sequence identity (10% for the head domain, 7% for the phospholipid binding domain**), additional structural predictions would significantly strengthen this conclusion. I recommend using AlphaFold predictions for C05G5.2 compared to human and Drosophila FAM21 to determine if the predicted folds are more conserved than the sequences. Furthermore, ColabFold analysis to predict conserved protein-protein interactions, particularly with other WASH components, Retromer, and capping proteins. Additionally, once C05G5.2 is established as WSHC-2, the authors should use "WSHC-2" consistently throughout the manuscript rather than waiting until page 6 to adopt this nomenclature. The gene name "C05G5.2" need only appear once upon introduction.
Response: We thank the Reviewer for these excellent suggestions. We have added:

a) AlphaFold predictions to show low identity in the head domain hides high similarity in predicted structure, including a long alpha helix that AlphaFold predicts with "very high confidence"(Fig. 4C);
b) ColabFold predictions, comparing *C. elegans* WSHC-2 to human and fly in revised Fig. 4, to show *C. elegans* WSHC-2 assembles into a similar structure with WSHC-5 (Fig. 4D), and quite importantly, the *C. elegans* CPI is predicted to bind to the stalk of the Capping Protein CAP-β/CAP-2, quite similarly as in human and Drosophila (4E);
c) We switch to WSHC-2 after Figure 4.

NOTE: We also tried ColabFold predictions for how *C. elegans* WSHC-2 and its orthologs would bind to Retromer component VPS-29 but ColabFold was not able to predict the known biochemical interactions and the crystal structure between specific LF motifs and Retromer component VPS-29 (Moreno-Romano et al., 2024 doi.org/10.1002/pro.4980).

3. In the Methods section, the "C. elegans strains built for this paper" section is inadequate. The authors list strain names but provide no information on construction methods. How were fluorescent tags inserted? (CRISPR/Cas9, MosSCI, other method?) What are the relevant guide RNA sequences and/or primers used?
Response: All of the strains "built for this paper", used strains that we had built previously, or that others had built previously, so the information for how they were made is already published. We now cite the "Strains used in this paper" first, and made sure to include the citations. We added that only two were made from strains with endogenous tags introduced using CRISPR, and that the rest are published multicopy arrays that were inserted into the genome, all previously published (page 7).

Minor point:
The autofluorescence subtraction method is not fully described in the methods. What kind of

thresholding and machine learning did you use? Is this useful for others, and can it be made available?

Response: We have expanded the description of the IMARIS methods used to subtract the autofluorescence. Please note we previously included information in the Figure Legend for Figure 3 and in the Methods. To make it easier for people to find the information, we have moved all of the descriptions to the expanded Methods section under the heading "Subtraction of autofluorescence and colocalization calculations," page 8.

"Subtraction of autofluorescence and colocalization calculations.
To calculate colocalization of red and green channels, we used the algorithm object-to-object to generate 3D surfaces rendered based on signal intensity in the red, green and blue channels. We manually adjusted the intensity thresholds to include the expected puncta, and used the Classification tool that includes a machine learning (ML) component to train the software to recognize red and green signal that overlapped with the blue channel, and then removed red and green surfaces that overlapped with the blue surfaces. The red and green surfaces were then analyzed for colocalization based on object-to-object statistics, specifically looking at the overlap volume ratio to surface and shortest distance to surface (surface edge to surface edge overlap)."

REVIEWER 1 ADDITIONAL COMMENTS

Review of Irizarry-Barreto et al., The *C. elegans* WASH complex supports epithelial polarity by promoting endosomal sorting of Cadherin.

Summary: Irizarry-Barreto et al. demonstrated that depletion of known components of the nucleation promoting factor (NPF) **WASH** complex, wshc-5, in *C. elegans* reduced the **localization of E-cadherin/HMR-1 at apico-lateral junctions in the adult intestine**. *wshc-5* depletion also **altered membrane trafficking**; specifically, there were significantly fewer **early endosomes (based on RAB-5**) and **recycling endosomes (based on RME-1**) and significantly **more late endosomes (based on RAB-7) and more Golgi (based on AMAN-1**). **The relative intensity of the proteins used to identify different endosomes was also found to significantly vary upon *wshc-5* depletion**. WSHC-5 was found to **co-localize with all endosomes and the Golgi** apparatus to some degree, showing the **most co-localization with early endosomes and RME-1 positive recycling endosome**s. To further investigate the role of the WASH complex in E-cadherin/HMR-1 trafficking, the authors investigated the co-localization between E-cadherin/HMR-1 and endosomes. They found that E-cadherin/HMR-1 was primarily co-localized with early endosomes, late endosomes, and RME-1 positive recycling endosomes. When *wshc-5* was depleted, they found a significant decrease in the colocalization between HMR-1 and early endosomes (RAB-5) but a significant increase in colocalization between HMR-1 and late endosomes (RAB-7), Golgi (AMAN-2), and some recycling endosomes (RME-1 & RAB-10). The authors further **propose that the uncharacterized protein, C05G5.2**, is a highly divergent ortholog of the WASH component, **FAM21/WSHC-2.** Consistent with this hypothesis, depletion of *c05h5.2* phenocopied depletion of other members of the WASH complex (wshc-5, ddl-1/wshc-3, or ddl-2/wshc-1) in **reducing apical F-actin, increasing apical and basal phospholipid distribution, and in altering the intensity of early endosomes (RAB-5**). While this work advances the field by characterizing WASH function in the adult *C. elegans* intestine and complements previous research by this group on the role of WAVE in E-cadherin/HMR-1 localization, I believe that additional experiments, including repetition of several experiments to increase samples sizes are needed to increase confidence in the conclusions.

Major concerns:

1) Figure 1 and associated text:

a. Sample sizes are not included for any of the experiments shown in Figure 1. These should be specifically mentioned in the figure legend.

Response: We have expanded the explanations for sample sizes in each Figure Legend. For the scatter plots, including new data added to Figure 1, we specify what each dot represents.

b. The final paragraph of the "*C. elegans* WASH complex supports apical enrichment of Cadherin" section makes claims that are not supported by the experiments.

The authors should conclude that WASH is required for maintenance of actin levels at the apical surface. There are no experiments performed that show that loss of E-cadherin/HMR-1 is responsible for the decrease in actin levels at the apical surface following depletion of the WASH components and the authors should revise the language "To directly visualize the consequences of Cadherin apicolateral loss" and "Thus, the decrease in Cadherin caused by depleted WASH complex has consequences for epithelial polarity of the adult intestine." Given that the authors have previously shown that depletion of *hmr-1* reduces actin levels, they could instead cite their paper and speculate that the reduction in *hmr-1* as a result of depleted WASH components is responsible for the changes they observe, and speculate that the changes in actin are indirectly caused by depleted WASH components. I would also strongly recommend softening the language about epithelial polarity in the final sentence. While actin levels are reduced, actin is still polarized and correctly localized. Other classical apical polarity proteins such as PKC-3 and PAR-6 are not examined, and the apical PIP2 and apically enriched PIP3 still appear to be apically enriched in the *wshc-5* and *wshc-2* RNAi treatments shown in figure 4, suggesting that the apico-basolateral polarity axis is largely preserved with the depletion of WASH components.

Response: We have softened the language and the claims as suggested by the Reviewer, page 3, we cite our own papers showing that loss of E-Cadherin reduces apical F-actin (page 3). In addition, we added data to Figure 1, I and J, showing that loss of the WASH components reduces apical F-actin levels.

2) Figure 2 and associated text:

a. The samples sizes are listed as a range from 3-8 animals. Specific sample sizes for each experiment should be listed. Additionally, a sample size of 3 seems too small, particularly as it should be relatively straightforward to obtain additional animals with RNAi feeding of young adults.

Response: We revised the Figure legends to document the sample sizes. Additional measurements were done on the RFP::RAB-10 movies, which had a small number in the original draft. The new data was added to Fig. 2. All Figures now list the number of animals. We moved some data from Supplemental to Revised Figure 3, so that both the number of animals and the thousands of endosomes included in the calculations can be more easily viewed.

b. The authors should quantify differences in endosomal localization alluded to in the second paragraph of the section, "WASH supports cellular organization of endosomal organelles". For instance, the authors state, "RFP::RME-1 is most enriched in basolateral regions and those regions showed the largest change".

This conclusion is not obvious and should be quantified. Similarly, changes in apical vs cytoplasmic RAB-11 and basal AMAN-2 should be quantified. There may be some additional changes in endosomal localization based on the authors images that may also be worth quantifying to support the author's argument that WASH is involved in epithelial polarity. E.g. the RAB-7 positive late endosomes look like they might be localizing to junctions in the *wshc-5* RNAi treatment and the RME-1 positive recycling endosomes might not be localizing to junctions in the *wshc-5* RNAi treatment.

Response: We revised this section, and after doing more measurements we have added measurements of the cytoplasmic signal in the RFP::RAB-7, RFP::RME-1 and RAB-11::GFP, phenotypes where there is a significant shift in the intensity of the puncta. The only endosomes showing a big shift to the cytoplasm were the RFP::RME-1 endosomes. For RFP::RAB-7, though the endosomes are brighter and the numbers are slightly elevated, there was no significant shift to the cytoplasm. RAB-11::GFP was lower everywhere, at the endosomes and at the cytoplasm.

Therefore, these data show shifts in endosome organization, but it was not clear that the shifts of the endosomes neatly align with shifts in polarity. The effect on polarity is more likely due to the E-Cadherin cargo being missorted, so that reduced total amount of E-Cadherin is able to reach its normal destination.

3) Figure 3 and associated text:

c. It is unclear what exactly is quantified in part C. Are the green dots showing the % of all the WSHC-5 in the ROI that is co-localized with each of the endosomal markers? Are the pink dots showing the % of all the endosomes that have WSHC-5 colocalized? What does "Total Compartment" mean. The key doesn't seem to match the figure legend.

Response: We agree these are confusing. We expanded the Figure Legend to clarify that 3C. is showing two kinds of data: Green dots show % of endosomes colocalizing with WSHC-5 out of all the visible WSHC-5, magenta dots show % endosomes colocalizing with WSHC-5 out of all the endosomes or Golgi. We originally labeled it "Compartment" instead of endosomes, since the Golgi are not considered endosomes. However, "Endosomal Organelles" may work better, since in the text we refer to "endosomal organelles". In the Figure Legend we explain, "Organelles" refers to endosomal organelles and the Golgi.

We heavily revised Figure 3, including moving the Table previously in Supplemental data into Fig. 3F, to support 3E. The Table shows how many endosomes are included in the data in the 3E graph, and the number of animals used for the IMARIS renderings and colocalization analysis. For the graph in 3C, we tried adding a supporting Table but Figure 3 is crowded as is. Therefore the Table that supports the graph in 3C is now in Supplemental Figure S1.

d. In part D, the Rab-11 RE colocalization with E-cadherin/HMR-1 is shown as significant at $p<0.0001$, but looking at the means/medians and the error bars, I would be surprised to find any difference between the control and experimental groups

Response: We reviewed the raw data and revised 3D, to include numbers for overlap of WSHC-5 and E-Cadherin out of total Cadherin puncta. For two data sets, RAB-11 and AMAN-2, we had mistakenly included overlap out of total endosomal puncta, which is noisier data.
In the revised Fig. 3D, we show the averages for individual worms, which show significant differences only for RAB-5, and RME-1 endosomal organelles. RAB-11 is not significantly different, and neither are RAB-7, RAB-10 or AMAN-2. In the new Table, Fig. 3F, the statistical test done in Prism, Grouped Analysis, using row statistics and multiple unpaired t test, generated significance values that make better sense.

e. Part E is not helpful for understanding differences in E-cadherin/HMR-1 localization between the control and experimental groups. I can't see any obvious differences among the images. I found Table 1 in the supplemental far more compelling for changes in E-cadherin co-localization particularly as E-cadherin levels are decreasing at the junctions, but significantly increasing in some endosomal compartments after the same period of treatment.

Response: Thank you for this feedback, which we used to revised Figure 3. Since Figure 3E was too busy and hard to appreciate, we moved the closeups from Fig. 3E into the Supplemental Data, Fig. S1. That left more room to enlarge the images in Fig. 3E to show how often the green and magenta signal overlap. In addition, we moved Table 1, previously in the Supplemental data, into Figure 3, now Fig. 3F. The Table lists the number of both animals compared and total endosomes compared. We think this makes it easier to see, in the images, graph and Table, that in some cases loss of WASH strongly affected green/magenta overlap, and in other cases it did not.

f. Specific sample sizes for each experiment should be included. For the co-localization analyses, the total number of ROI per worm should also be specified.

Response: We have added the exact sample size since 3F includes this information in the Table.

g. For the graphs, the bars should be explain. Is the middle bar the mean or median? Are the vertical bars representing Standard Deviation, Standard Error of the Mean, or something else?

Response: We expanded the explanation in the Fig. 1 legend to state, "Here and in all figures, the central lateral bar shows the Mean, while vertical bars show 95% confidence intervals." (page 13).

h. At the end of the last paragraph in the section, "WASH is enriched at some endosomal organelles" the authors refer to observations made in the 2nd author's Master's thesis. Since Master's theses are not subject to peer review, this data may be better published as supplementary data in this publication. Or, the authors should conduct similar experiments to determine if blocking lysosomal degradation would also restore levels of E-cadherin/HMR-1.

Response: : We agreed. We conducted new experiments and added the data to Figure 1, to show that: a) we can rescue loss of Cadherin in a WSHC-5 depleted animal by blocking lysogenesis with the *cup-5* mutant (Fig. 1 G,H); b) Loss of WASH components, just like loss of WAVE components, reduces apical F-actin in the intestine (Fig. 1I,J).

We were going to completely remove the Master's Thesis, except that we keep finding evidence that other scientists are finding it, and using it to guide their experiments. We don't rely on any data from the Master's Thesis, but we mention it exists and added an updated link that should be accessible to all.

3) Figure 4 and associated text:

a. C05G5.2 is so divergent from the Human and *Drosophila* FAM21 proteins, that stronger evidence is needed to establish this protein as an ortholog. How was C05G5.2 identified as a likely WSHC-2/FAM21 ortholog? How were sequences aligned? The argument that C05G5.2 is the WSCH-2/FAM21 ortholog would be further strengthened by completing one of the following experiments: 1) Use CRISPR-Cas9 to endogenously tag C05G5.2 and determine colocalization with WSCH-5 or 2) a Yeast two hybrid experiment to determine if there are physical interactions between C05G5.2 and WSHC-5 or between C05G5.2 and CAP-1 and CAP-2 or 3) a pull-down assay to show interaction between C05G5.2 and other WASH and CAP proteins.

Response: We thank the Reviewer for these excellent suggestions. We have added AlphaFold alignments, and ColabFold predictions and include this now in Revised Figure 4.
We were not able to tag WSHC-2 in time to add colocalization studies, but we are still trying.

b. In part D, why is there such a large difference in RAB-5 basal intensity between the control used for the *wshc-5* depletions and the control used for the *wshc-1* and *c05G5.2* depletions?

Response: Those experiments were done at two times due to microscope breakdown issues. Now that the microscope is back up and running, these experiments were repeated, so all phenotypes could be compared at the same time. The revised results were moved to Revised Fig. 5A.

Minor concerns:

Figure 1 and associated text:

A. In part A make sure that the order of the protein names is consistent on the cartoons of the complexes, with the *C. elegans* name always last (or first), but keep it consistent.

Also, the names are difficult to read when they are not entirely within the rounded rectangles that represent the proteins.

Response: In the Revised Figure 1A, we increased the size of the smallest members of each complex and reduced the font size so we could consistently list Human/Worm names.

B. Label the E-cadherin and (branched actin? in red) on the figure just as all of the other complexes are labeled.

Response: Thank you for catching this! The labels for E-cadherin and branched actin were removed by mistake and are now added back.

C. In part F, is WASH expected/predicted to be at all of the endosomes and Golgi or only in retromer transport, from early endosomes to the Golgi as listed in the figure legend.
From the cartoon alone, it is unclear where WASH is expected to play a role.
Response: From the published literature, WASH was expected to work at early endosomes, sorting cargo with the help of Retromer. We say this on page 3, when we cite Fig. 1F, "WASH is expected to be enriched at endosomes (Derivery et al., 2009; Gomez and Billadeau 2009; Romano-Moreno et al., 2024, Fig. 1F)."

D. In the second paragraph of the "*C. elegans* WASH complex supports apical enrichment of Cadherin" section, the authors write "Depleting *wshc-5*/*Strumpellin* for 24 hours showed mild changes….36 hour depletion showed stronger changes, while 48 hour depletion strongly reduced Cadherin/HMR-1…" It would help the readers to have those changes more specifically identified as reductions in E-cadherin/HMR-1 instead of general "changes".
Response: We changed the wording to say, "reductions in junctional accumulation of E-Cadherin/HMR-1…."

E. In the figure legend for figure 1, the frequency of the word "most" leads me to wonder when the authors looked at intestinal rings other than Int2/Int3 and which images were not at an apical focus. Since all the images are apical focuses (I think at Int2/Int3) until figure 4, I would recommend removing "most" from the description. (I also think the phrase "…making live imaging simple" is unhelpful to most readers as non-*C. elegans* or *C. elegans* researchers not focused on studying the intestine won't know why Int2/Int3 are simple to image and the researchers who do study *C. elegans* intestine don't need the explanation.) Finally, I think it would be helpful to identify the statistical tests performed and significance in each of the figure legends instead of only in the Figure 1 legend.
Response: We changed the wording to say "Images in most figures are shown from an apical focus, unless otherwise stated, which allows comparison of protein enrichment at apical lumen, apicolateral (red arrows), and basolateral (blue arrows) membrane regions." We imagined non-*C. elegans* readers might be confused why only a tiny region of the intestine was imaged. For the Statistical Tests, we used the same one-way ANOVA for everything, except for Fig. 2E, where we used a Student T test with Welch's correction."

Figure 2 and associated text:

A. I don't think the cartoon in part A is necessary as the same orientation and apical views were used as were already shown in figure 1.
Response: We removed the cartoon from Figure 2A.

B. Statistical tests and significance values should be included.
Response: This was done.

Figure 3 and associated text:

A. I don't think the cartoon in part A is necessary as the same orientation and apical views were used as were already shown in figure 1. I also don't think the embryo image is necessary the embryonic puncta were previously described.
Response: We decided to leave this. It shows how different the analysis is (and how much harder) in a complex tissue like the adult intestine. It also shows the same strain is useful for different tissues and times in development.

B. The WSHC-5 signal in the L4 intestine in Part A is very hard to see compared with the autofluorescence even when the image is enlarged on a computer screen. If possible, the WSCH-5 signal should be enhanced, even if that requires overexposure of the gut granules to help the reader see the signal without the yellow circles on the Surface – DAPI.

Response: Perhaps the Reviewer is starting to appreciate how difficult it is to image the adult intestine. This is why we were so careful about subtracting the autofluorescence. The early embryo is, by comparison, much easier to image.

C. DAPI is a specific dye used to see nucleic acids. The blue autofluorescence should be labeled as "Autofluorescence" or "Gut granules" instead of "DAPI" to avoid confusion.

Response: We changed "DAPI" to "AUTO" and explain in the Figure legend AUTO = autofluorescence caused by the gut granules.

D. In part B of Figure 3, are the top 2D max projection images and the 3D surface rendered projections supposed to be showing the same ROI in FIJI and in Imaris? They do not appear to be showing the same region, so some clarification would be useful.

Response: We revised Fig. 3B to show the raw data, with three channels (magenta, green, blue) and also just magenta and green, to emphasize how much of the larger green structures are likely autofluorescent gut granules. The crops are not the same, explaining the altered views. To make this more clear, we added two sets of BLUE dotted lines to 3A: the smaller "B" box is the region that is shown in 3B, which a larger box applies to the intestinal regions shown in 3A and 3D, which is at the Ring 2/3 region, for the full width of the worm.

E. In the figure legend for part B, "…mNG::WSCH-5 in Cyan" should be corrected to "…in green".

Response: Thank you. It has been changed to "green".

Figure 4 and associated text:

A. A basal intestinal view is shown for the first and only time in figure C. Here it would be helpful to bring back your cartoon to show the difference between the apical and basal views.

Response: Thank you for the suggestion. Figure 4 was heavily revised, so the cartoon was not needed there. This basal data was moved to Fig. 5A, with labels for the Basal and Apical views. We also included dotted lines to emphasize that these are two different views of the same tissue.

B. Since basal views are sown in C, it would be helpful to clarify that apical views are shown again in E.

Response: These results were moved to Fig. 5A, and we added, Top row: Basal view, dotted line shows outline of the intestine; Bottom row: Apical view, dotted lines show apical lumen.

Supplemental Figure 1:

A. It would be helpful to show the post autofluorescence subtraction images as well to see what was ultimately quantified.

Response: These images were added back to Fig. 3B, and removed from Fig. S1. The Figure Legend explains that those are the images shown in Figure 3B, the top two rows.

Other minor comments:

A. The master's thesis which is not subject to peer review is referenced in the introduction for the paper. It may be better to include the evidence from the master's thesis either as part of one of the main figures or as a supplemental figure instead of referring to the thesis.

Response: We include new experiments in Fig. 1G,H,I and J, so we do not refer to experiments from the Master's Thesis. An updated link to the Thesis was added, since people are using it.

B. While HMR-1 is the only classical Cadherin in *C. elegans,* worms do have 13 total cadherins. Additionally, for non-*C. elegans* researchers who read this work, I think it would be helpful to clarify E-cadherin instead of just referring to HMR-1 as Cadherin throughout the text.

Response: Yes, that is a good point, and not minor. We went back and changed "Cadherin" to "E-Cadherin" throughout, including in the title.

RE: Manuscript #E25-12-0612R
TITLE: "The C. elegans WASH complex supports epithelial polarity by promoting endosomal sorting of E-Cadherin"

Dear Dr. Soto:

I am pleased to accept your manuscript for publication in Molecular Biology of the Cell.

Sincerely,
Joshua Kelley
Monitoring Editor
Molecular Biology of the Cell

Dear Dr. Soto:

Congratulations on the acceptance of your manuscript! Thank you for publishing your work in Molecular Biology of the Cell (MBoC).

Within 10 days, an unedited PDF of your manuscript will be published on MBoC in Press, an early-release journal version. The date your manuscript appears on this site is the official publication date.

Your copyedited and typeset manuscript will be scheduled for publication in the next available issue of MBoC. Once your paper is ready for review, our production team will notify you. In the summer of 2024, our production provider will introduce an online proofing solution, which will streamline the process of checking and validating author changes. This will result in improved typesetting quality, enhanced digital content, and a faster overall process.

We invite you to submit images related to your accepted manuscript to be considered for the journal cover.
Cover images must contain color, TIF Format
Size image at 7.4" wide x 5.85" deep
RGB and CMYK color (please submit two separate files, one in each format)
300 dpi resolution

Please also send a 1-paragraph description of the image that a general audience could understand. Include with the caption the name and institution of the individual(s) the image should be attributed to. Please provide this description in a Word file. You can send the file via email or by your favorite file transfer service to mboc@ascb.org.

Authors of Articles and Brief Communications are also encouraged to create a short video abstract to accompany their article when it is published. These video abstracts, known as Science Sketches, are up to 2 minutes long and will be published on YouTube and then embedded in the article abstract. Science Sketch Editors on the MBoC Editorial Board will guide you as you prepare your video. Information about how to prepare and submit a video abstract is available at www.molbiolcell.org/science-sketches. Please contact mboc@ascb.org if you are interested in creating a Science Sketch.

We look forward to publishing your paper in MBoC.

Sincerely,

Eric Baker
Journal Production Manager
MBoC Editorial Office
mbc@ascb.org
